# Fabrication and Characterization of Biodegradable Gelatin Methacrylate/Biphasic Calcium Phosphate Composite Hydrogel for Bone Tissue Engineering

**DOI:** 10.3390/nano11030617

**Published:** 2021-03-02

**Authors:** Ji-Bong Choi, Yu-Kyoung Kim, Seon-Mi Byeon, Jung-Eun Park, Tae-Sung Bae, Yong-Seok Jang, Min-Ho Lee

**Affiliations:** 1 Department of Dental Biomaterials, Institute of Biodegradable Materials, School of Dentistry, Jeonbuk National University, Jeonju-si 54896, Jeollabuk-do, Korea; submissi@naver.com (J.-B.C.); yk0830@naver.com (Y.-K.K.); pje312@naver.com (J.-E.P.); bts@jbnu.ac.kr (T.-S.B.); 2 Dental Clinic of Ebarun, Suncheon-si 57999, Jeollanam-do, Korea; sumse1205@naver.com

**Keywords:** hydrogel, tissue engineering, biphasic calcium phosphate nanoparticle (BCP-NP), biocompatibility, biodegradable, gelatin methacryloyl (GelMA), visible light

## Abstract

In the field of bone tissue, maintaining adequate mechanical strength and tissue volume is an important part. Recently, biphasic calcium phosphate (BCP) was fabricated to solve the shortcomings of hydroxyapatite (HA) and beta-tricalcium phosphate (β-TCP), and it is widely studied in the field of bone-tissue engineering. In this study, a composite hydrogel was fabricated by applying BCP to gelatin methacrylate (GelMA). It was tested by using a mechanical tester, to characterize the mechanical properties of the prepared composite hydrogel. The fabricated BCP was analyzed through FTIR and XRD. As a result, a different characteristic pattern from hydroxyapatite (HA) and beta-tricalcium phosphate (β-TCP) was observed, and it was confirmed that it was successfully bound to the hydrogel. Then, the proliferation and differentiation of preosteoblasts were checked to evaluate cell viability. The analysis results showed high cell viability and relatively high bone differentiation ability in the composite hydrogel to which BCP was applied. These features have been shown to be beneficial for bone regeneration by maintaining the volume and shape of the hydrogel. In addition, hydrogels can be advantageous for clinical use, as they can shape the structure of the material for custom applications.

## 1. Introduction

Currently, the most common method for repairing damaged bone tissue is to implant bone-graft material directly into the defect. Additionally, in order to recover the defective area in the clinical environment, bone tissue is regenerated, using guided bone regeneration (GBR), and then treatment through implants is performed. Clinically available bone-graft materials are often provided in powder form. These powdered implants are mixed with saline or blood, to maintain their shape. The powdered bone-graft material can be easily applied to small bone defects. However, In the case of large defects, it is difficult to maintain the shape, due to weak adhesion by blood or saline solution. In these situations, auxiliary materials such as metal (titanium, magnesium, etc.) mesh are used to maintain shape. However, there are still some limitations related to the prolongation of the infection and recovery period after treatment and the delivery of biological mediators for tissue regeneration [1,2]. Membranes based on natural hydrogels show great potential in tissue-engineering applications, due to their tunable physical properties. In addition, the natural hydrogel-based membrane is in the spotlight, as an alternative for the delivery of various controlled substances in vivo due to micro-level control, increase in mechanical properties, and control over decomposition rate. For example, a study [3] on dental regeneration by encapsulating cells fabricated by using a dentin-derived hydrogel was reported. Another study [4] reported the development of hydrogels based on photopolymerized gelatin containing cells by visible light (VL), using a dental transition device. Gelatin methacryloyl (GelMA), manufactured by substituting natural polymers, is a compound synthesized by adding a methacrylate group to the amine and hydroxyl groups of gelatins. However, in addition to the biocompatibility and expansion ratio, which are basic characteristics of gelatin, it has a motif that is degraded by RGD peptide (arginine-glycine-aspartic acid) and Matrix Metalloproteinase (MMP) related to cell adhesion [5,6]. Gelatin-methacrylic anhydride (GelMA), in which a methacrylate group is added to the gelatin amine group, can be used to manufacture a hydrogel by photocrosslinking. GelMA hydrogel using photo-crosslinking showed good biocompatibility in the body, due to its high biological properties [7]. In addition, it is widely used in tissue engineering, through cell culture and drug delivery, in consideration of properties similar to the extracellular matrix (ECM) [8,9]. However, compared to other polymers, such as alginate and polylactic acid hydrogel, the mechanical properties are significantly lower [10,11,12]. Therefore, in order to apply GelMA to bone-tissue regeneration, it is necessary to improve the mechanical strength by adding other materials. GelMA nanocomposites were developed by using synthetic bones, such as hydroxyapatite (HA) or β-tricalcium phosphate (β-TCP), and many studies have been conducted to improve bone function [13,14]. HA is known as the main component of bones and has high biocompatibility and strength, which complements the strength of other minerals (β-TCP). The composite material containing HA particles and HA showed good regeneration ability in the bone-defect model, but after degradation, HA remained in the regenerated site and could not be completely replaced by new bone [15]. Moreover, delayed biodegradation can interfere with bone remodeling [16]. On the contrary, β-TCP can be easily degraded in the body, activates bone conduction through micropores inside the body to form new bone, and is known to cause complete bone regeneration, because it does not remain in the tissue [17]. Biphasic calcium phosphate nanoparticles (BCP-NPs), which are a mixture of HA and β-TCP at 60/40 wt%, are known as a composite material that can compensate for the disadvantages of HA and β-TCP, which are synthetic bone materials [18]. Recently, a hydrogel system containing biphasic calcium phosphate has been studied for effective bone regeneration. Lei Nie et al. reported that BCP-NP-conjugated chitosan/gelatin hydrogel, combined with the bone marrow mesenchymal stem, exhibits an excellent bone formation reaction. Omar Faruq et al. demonstrated excellent osseointegration and improved bone formation by incorporating BCP-NP into hyaluronic acid/gelatin hydrogels [19,20]. However, further studies to confirm the effect of these hydrogel systems directly are required. In this study, we designed composite hydrogel after directly fabricating BCP-NP and combining it with the GelMA hydrogel. The composite hydrogel was chemically crosslinked by photopolymerization, using visible light, and formed a physical bond with BCP-NP. The prepared composite hydrogel was analyzed for physical and chemical properties, and biocompatibility was confirmed through an in vitro test.

## 2. Materials and Methods

### 2.1. Materials

All materials used in the synthesis of GelMA (Type A gelatin, Methacrylic anhydride (MA), dialysis tubing, high-retention seamless cellulose tubing (12–14 kDA MWCO, 40 mm diameter), Dulbecco’s phosphate-buffered saline (DPBS) Dialysis tubing closures), fabrication of hydrogel (Triethanolamine (TEA), Eosin Y disodium salt, N-Vinylcaprolactam (VC)), fabrication of biphasic calcium phosphate nanoparticles (BCP-NP) (calcium nitrate tetrahydrate (Ca(NO_3_)_2_·4H_2_O, and ammonium phosphate dibasic ((NH_4_)2HPO_4_) were purchased from Sigma-Aldrich (St. Louis, MO, USA) and used without further purification. Moreover, α-MEM (Minimum Essential Medium Eagle-α), 10% fetal bovine serum (FBS), and antibiotics (penicillin/streptomycin) used for the cell experiment were purchased from Gibco BRL (Invitrogen Co., Carlsbad, CA, USA). Cell proliferation and activity were evaluated, using CCKi-8 (Enzo Life Science Inc., New York, NY, USA) and a TRACP & ALP assay kit (TakaRa, Kyoto City, Kyoto Prefecture, Japan).

### 2.2. Equipment

To confirm the substitution of GelMA, a nuclear magnetic resonance (1H-NMR) spectrum was recorded, using JNM-ECZ600R (JEOL, Akishima City, Tokyo, Japan) installed in the Center for University-Wide Research Facilities (CURF) at Jeonbuk National University. Fourier transform infrared spectroscopy (FTIR, Frontier (Perkin Elmer, Waltham, MA, USA)) was used to measure the molecular bonding structures and spectroscopic properties of hydrogels and other materials. The crystal phase of BCP-NP and polydopamine contained in the hydrogel was analyzed qualitatively and quantitatively. This was graphically plotted by using X’PERT-PRO Powder (PANalytical, Malvern, British). The crystal phase of BCP-NP was observed, using a Bio Transmission Electron Microscope (H-7650, HITACHI, Marunouchi, Chiyoda-ku, Tokyo, Japan), at a magnification of 100,000–200,000, with 100 kV of acceleration voltage. The hydrogel morphology was analyzed, using a Field-Emission Scanning Electron Microscope (FE-SEM; SU-70, HITACHI, Japan) and energy-dispersive X-ray (EDX) spectrometer (OCTANE PRO, AMETEK^®^, Burwin, PA, USA). The mechanical properties of the hydrogel were evaluated by using a universal testing machine (Instron 5569, Instron, Norwood, MA, USA), and cell proliferation and activity were evaluated, using an ELISA reader (Molecular Devices, EMax, San Jose, CA, USA). Additionally, thermogravimetric analysis was evaluated by using SDT Q600 (WATERS, Milford, MA, USA).

### 2.3. Synthesis of GelMA and BCP-NP

GelMA was synthesized by previously reported methods [8,21], using gelatin type A. Briefly, 10% (*w*/*v*) of gelatin was added to DPBS and dissolved completely at 60 °C. Then, MA (0.8 mL MA to 1 g gelatin ratio) was added to the gelatin solution and stirred at 50 °C for 3 h. The reaction was terminated by diluting the solution 5 times by volume. This was followed by dialyzing for 1 week, using a 12–14 kDA cutoff dialysis pack in deionized water, at 40 °C. The dialyzed GelMA solution was freeze-dried, at −78 °C, for 5 days, and the resulting white GelMA foam was stored in a freezer, at −20 °C, before use. The preparation method for BCP-NP is as follows [22]. First, 75.0 mL of 0.5 M calcium nitrate tetrahydrate (Ca (NO_3_)_2_·4H_2_O, Sigma-Aldrich (St. Louis, MO, USA)) solution was slowly added to 50 mL of 0.5 M ammonium phosphate dibasic ((NH_4_)_2_HPO_4_, Sigma-Aldrich (St. Louis, MO, USA)). The mixture was adjusted to a 9.5 pH and allowed to react at 55 °C for 30 min. The resulting slurry was aged for 40 h at 25 °C, to form stable BCP-NP. Then, BCP-NPs were centrifuged, freeze-dried, and stored in a freezer, at −20 °C, before use.

### 2.4. Preparation of GelMA/BCP-NP Composite Hydrogel

**Figure 1:**
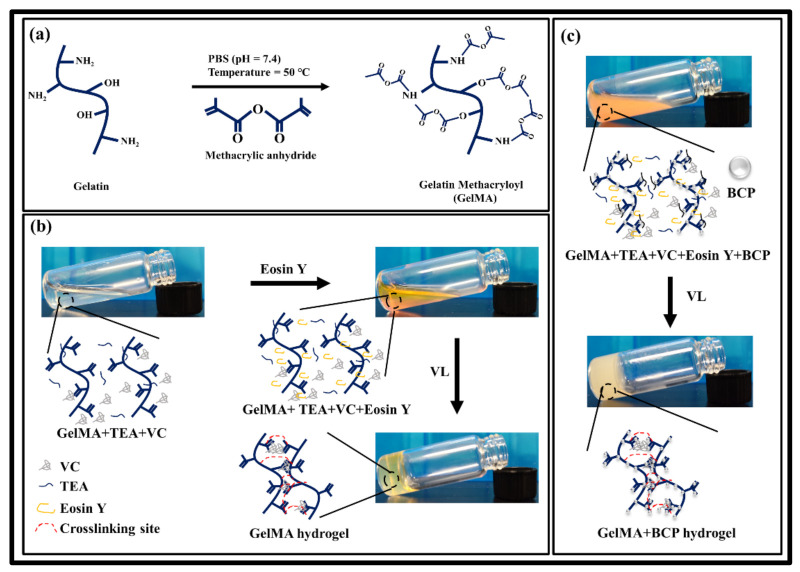
Schematic diagram of network structure formation on (**a**) gelatin methacryloyl, (**b**) gelatin methacryloyl hydrogel, and (**c**) biphasic calcium phosphate nanoparticles (BCP-NPs)-bonded gelatin methacryloyl hydrogel.

GelMA hydrogel was fabricated by visible (VL), using photopolymerization with Optilux Demetron curing light (Kerr Inc., Danbury, CT, USA). In distilled water containing TEA 1.88% (*v*/*v*) and VC 1.25% (*w*/*v*), different concentrations of freeze-dried GelMA (7%, 15% (*w*/*t*)) were dissolved. Eosin Y sodium salt with 0.05 mM concentration was prepared separately in distilled water. The TEA/VC solution containing GelMA (7%, 15% (*w*/*t*)) was mixed with Eosin Y, before crosslinking to form the final precursor solution. The precursor solution prepared to form hydrogel was pipetted at 1 mm intervals between two glass slides and exposed to visible (VL) for 120 s, at a distance of 5 cm. The GelMA/BCP-NP mixture was prepared as a composite hydrogel, through the same process as above. Briefly, BCP-NP was added to the GelMA aqueous solution containing TEA/VC. After that, ultrasonic treatment was performed for 2 h, to uniformly disperse BCP-NP in the solution. The dispersed solution was mixed with Eosin Y, to carry out the photopolymerization. The final precursor solution formed polymerization by visible light, for 120 s, to form a composite hydrogel. The resulting hydrogel was labeled GelMA-XX, according to the concentration of GelMA. BCP is labeled as GelMA-XXBYY, according to its weight (example: GelMA-7, GelMA15, GelMA15B0.1, and GelMA15B10). The prepared samples were stored in a refrigerator, at −20 °C, until use. A schematic diagram of the synthesis of GelMA, based on the reaction of gelatin with methacrylic anhydride and photo-initiator, is shown in Figure 1.

### 2.5. Characterization of BCP-NP and Composite Hydrogel

To confirm the crystal phase of samples were freeze-dried for 1 day and cut into the appropriate sizes, for analysis, by FE-SEM, FTIR, and XRD. FTIR analysis was performed within the wavelength 4000–500 cm^−1^ (KBr), using the attenuated total reflectance (ATR) method. BCP-NP and fabricated hydrogels were analyzed by XRD in a 20–60° range, with a 2° per minute gap at 60 kV. For FE-SEM, samples were sputter-coated with platinum and measured under an acceleration voltage of 10 kV.

### 2.6. Mechanical Analysis of GelMA Composite Hydrogel

For compression tests, GelMA and GelMA composite hydrogels of varying compositions were prepared as cylindrical samples with a 15 mm diameter and 2 mm thickness, which were measured by using a universal testing machine (Instron 5569). Hydrogels used for the measurement were hydrated in deionized water, for 24 h, and the experiment was performed at the maximum hydration condition. For accurate measurement, the sample was fixed between two parallel plates, and a 500 N load cell was mounted until fracture at a speed of 0.5 mm/min. The measured data were obtained, using Bluehill 2 software, and the modulus of elasticity was calculated at the first 10% strain of the curve in the stress–strain curve.

### 2.7. Hydrogel Degradation Rate Evaluation

The evaluation of the degradation rate of the prepared hydrogel and composite hydrogel was performed according to the following method [11]. Then, 25 U/mL^−1^ of collagenase (TypeⅡ, Worthington Biochemical Co.) was prepared in PBS, 0.5 mL each was added to the hydrogel sample, and then gently shaken on a shaker, at 37 °C. After that, the hydrogel samples were taken out at various times, and the dried weight was measured. The results are expressed as (M_t_/M_0_)^1/2^, where M_0_ is the initial weight of the hydrogel, and M_t_ is the dry weight measured at time, t.

### 2.8. Thermogravimetric Analysis of Hydrogel (TGA)

GelMA and composite hydrogels were used for TGA. Hydrogel was freeze-dried after freezing at −80 °C. The thermal behavior of the samples was carried out on an SDT Q 600 unit, with a 10 °C/min heating lamp, from 25 to 600 °C.

### 2.9. Cell Viability

Cell experiments were conducted in this study by using the MC3T3-E1 preosteoblast that was provided by ATCC (American Type Culture Collection). For the culture medium, 10% FBS of a nutrient component containing antibiotics was added to an α-MEM medium. Cell culture was performed in an incubator (3111, Thermo Electron Corporation, Waltham, MA, USA), at 37 °C, in a 5% CO_2_ atmosphere.

#### 2.9.1. Water-Soluble Tetrazolium Salt (WST) Assay

The proliferation capacity of MC3T3-E1 cells against the prepared composite hydrogel was measured, using a water-soluble tetrazolium salt (WST-8) assay (ALX-850, Enzo Life Sciences, Farmingdale, NY, USA). After a hydrogel is swelled to the maximum hydration, it was cut to a 10 mm diameter and placed in a glass bottle, followed by sterilization by autoclaving at 121 °C for 20 min. The sterilized sample was immersed in the medium for 1 h and then placed on the 12-well plate. The MC3T3-E1 cells were cultured for 3 and 5 days, at a cell density of 1 × 10^5^ cells mL^−1^. After 3 and 5 days, the medium was removed, and a 400 µL mixed solution containing water-soluble tetrazolium salt reagent and the α-MEM medium was dispensed and then stored in the 5% CO_2_ incubator. After 90 min, 100 µL was added to a 96-well plate, and the absorbance was measured at 450 nm, using the ELISA reader (Molecular devices, Emax, San Jose, CA, USA).

#### 2.9.2. Alkaline Phosphatase (ALP) Activity

ALP activity was evaluated, using the TRACP & ALP assay kit. The TRACP & ALP assay kit can detect the activity of acidic phosphatase (ACP) and alkaline phosphatase (ALP), respectively. In this study, it was used to detect ALP, an enzyme marker in osteoblasts. The cell culture method is the same as the method used in the WST assay. After 7 days of incubation, the differentiation of the preosteoblast cells was evaluated through the expression of ALP activity. The plates and composite hydrogels were washed, using saline. After that, the P-nitro-phenyl phosphate (pNPP) solution containing the ALP buffer solution and the extraction solution was added, following the kit protocol, and was allowed to react in the incubator, at 37 °C, for 1 h. After reaction completion, the ALP activity was evaluated by dispensing 100 µL of the solution in a 96-well plate and measuring the absorbance at 405 nm, using the ELISA reader.

### 2.10. Statistical Processing

Statistical analysis for all experimental results was conducted by using one-way analysis of variance with the Tukey test. A *p*-value lower than 0.05 was considered statistically significant (* *p* < 0.05, NS *p* > 0.05).

## 3. Results

### 3.1. Synthesis and Characterization of GelMA Macromers

The 1H-NMR spectra of gelatin and gelatin methacryloyl are shown in Supplementary Materials Figure S1. Compared with gelatin, derivatization by methacryloyl reaction was confirmed in the GelMA. In methacrylic anhydride, the shift of methacrylate protons was found at 1.8 ppm. In GelMA, a decrease in signal was observed at 2.9 ppm, due to the reaction of lysine amino acid and methacrylic acid. In addition, an increase in 5.4 and 5.7 ppm signals of gelatin methacryloyl was observed by the integrated lysine amino acid. The degree of methacrylate substitution (DOS) of GelMA was calculated by comparing the 2.9 ppm protons of unmodified gelatin and modified gelatin, and the calculated equation is as below (1) [23].

DOS = 1 − (lysine methylene proton of GelMA)/(lysine methylene proton of gelatin) × 100%(1)

### 3.2. Chemical Properties

**Figure 2:**
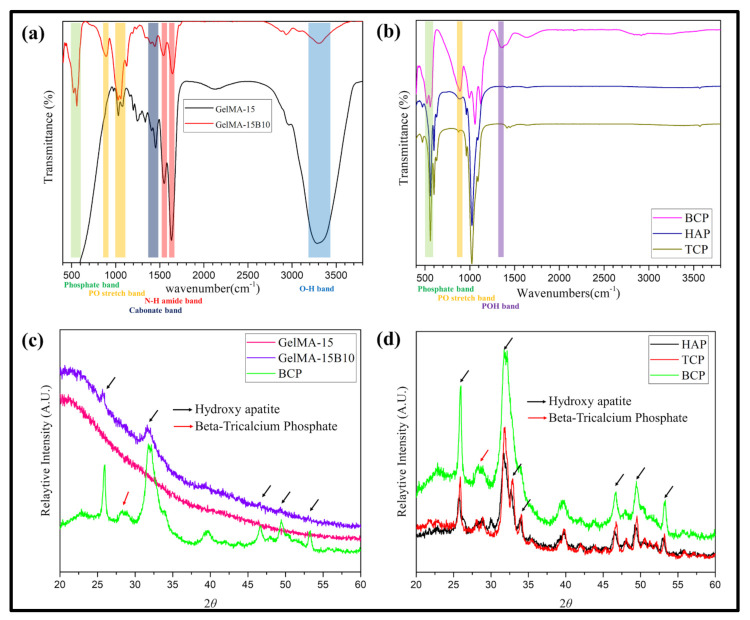
FTIR and XRD analysis of hydrogels (GelMA-15, GelMA-15B10, BCP-NP) and Nano-particles (BCP-NP, HAP, and TCP); FTIR; (**a**) hydrogels, (**b**) nano-particles/XRD, (**c**) hydrogels, and (**d**) nanoparticles.

The FTIR spectra of hydroxyapatite (HAP) and Tri-calcium phosphate (TCP) for comparison between gelatin hydrogel and composite hydrogel with BCP-NP and BCP-NP are shown in Figure 2a,b. In GelMA-15, the characteristic bands of hydrogels, such as NH amino band (1550 and 1650 cm^−1^), OH band (3200–3500 cm^−1^) and carbonate band (1425 and 1450 cm^−1^) were observed. In GelMA-15B10, it was confirmed that the intensity of the O-H band and N-H amino band decreased due to the incorporation of BCP-NP. In addition, an increase in the 1030 cm^−1^ band, which is a strong band assigned to the stretching of PO for HPO_4_^2−^ and PO_4_^3−^, and a newly generated 890 cm^−1^ band was shown. Compared to the HAP and TCP graphs, the graphs of different aspects could be confirmed in BCP-NP. A single phosphate band between 500 and 600 cm^−1^ is observed in HAP and TCP, but shows two well-decomposed phosphate bands in BCP-NP. In addition, only the BCP-NP was able to observe the band allocated to the stretching of PO (890 cm^−1^) and the characteristic band (1343 cm^−1^) caused by the bending of POH. The results of the XRD analysis for the hydrogel and BCP-NP are shown in Figure 2c,d. It was confirmed that the peak of the prepared BCP-NP coincided with the peak of the previous paper, and a mixed peak of HAP (25.9°, 31.8°, 46.7°, and 49.5°) and β-TCP (25.9°, 33.6°, and 53.2°) was observed [22]. The peak of BCP-NP was not observed in GelMA-15; only a hydrogel peak at a certain level was observed. In GelMA-15B10, both the peak of BCP-NP and the peak of hydrogel were included. In particular, all HAPs showing high peak values were observed, and no peaks of β-TCP, which are low peaks, were observed. In comparison between nanoparticles, most similar peak values were observed in BCP-NP, HAP, and TCP. However, the HAP and TCP (about 27° and 29°) peaks divided into two were merged into one, in the BCP-NP, showing a β-TCP peak, and it was confirmed that the HAP and TCP peaks observed between 34° and 35° disappeared in the BCP-NP.

### 3.3. Morphological Analysis

**Figure 3:**
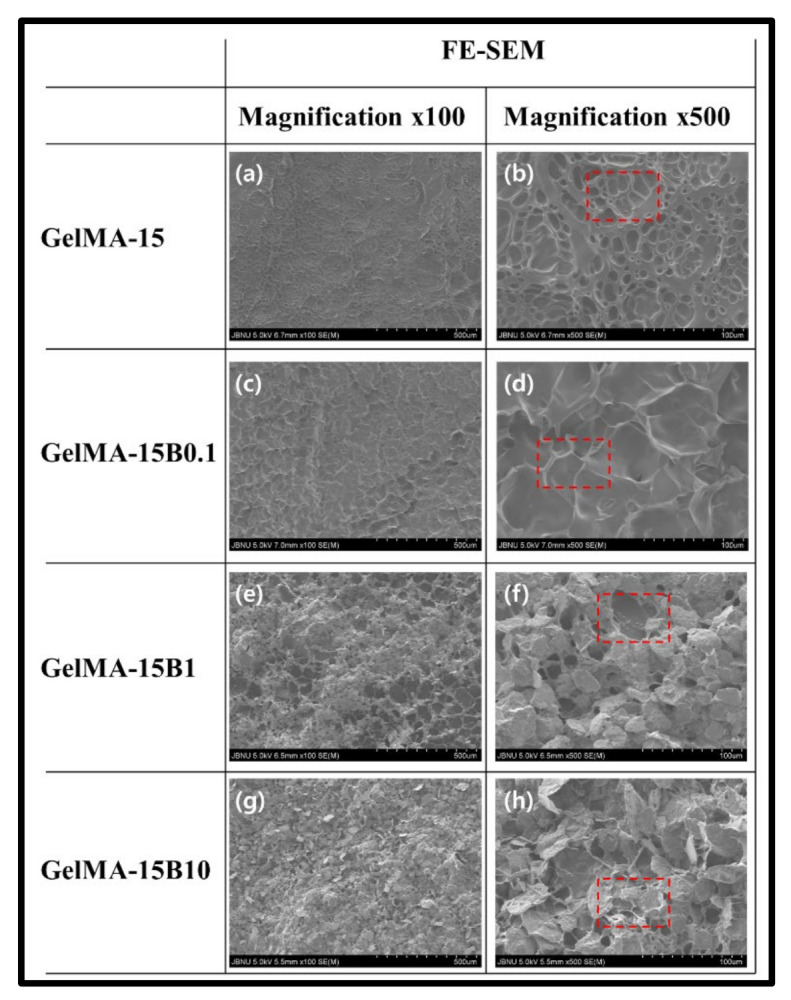
FE-SEM images on surface of (**a**,**b**) GelMA-15 and (**c**,**d**) GelMA-15B0.1, (**e**,**f**) GelMA-15B1 and (**g**,**h**) GelMA-15B10 hydrogels, at different magnifications (×100 and ×500). Red area: surface morphology according to BCP capacity increase.

The surface shape of the composite hydrogel containing BCP-NP and GelMA-15 is shown in Figure 3. The surface of GelMA-15 contracted by light polymerization did not show any pore shape and has an irregular structure in the form of a spider web (Figure 3a,b). GelMA-15B0.1 shows some regular surface shape, due to electrostatic attraction between hydrogel and BCP-NP due to the application of a small amount of BCP-NP (Figure 3c,d). In GelMA-15B1, it was possible to confirm the appearance that an appropriate amount of BCP-NP was covering the surface (Figure 3e,f). In addition, the BCP-NP was not evenly distributed on the surface and was able to confirm the sporadically clustered form. GelMA-15B10 was able to confirm that the entire surface was covered as an excess of BCP-NP was applied, and a more even surface was confirmed than for GelMA-15B1 (Figure 3g,h). EDX results for each hydrogel group are presented in Supplementary Materials Figure S2.

### 3.4. Mechanical Properties

**Figure 4:**
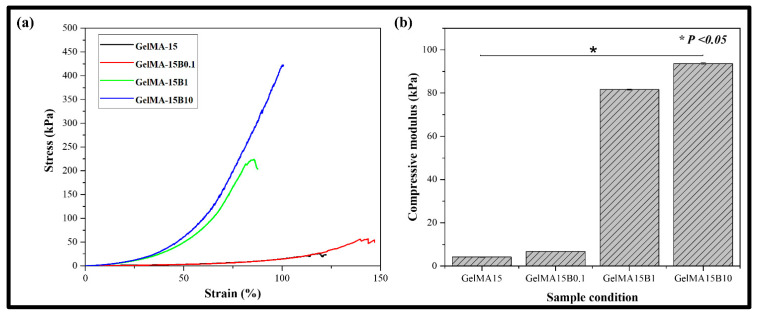
Graphs of (**a**) compressive stress–strain curve and (**b**) compressive modulus on the GelMA-15, GelMA-15B0.1, GelMA-15B1, and GelMA-15B10 groups.

It is known that a composite hydrogel containing BCP-NP can easily control its mechanical properties, according to the weight of BCP-NP [24]. For confirmation, a mechanical strength test was performed by varying the weight of BCP-NP (0.1, 1, and 10 mg). The stress–strain graph obtained through the compression test is shown in Figure 4a. GelMA-15B0.1 showed no significant difference between GelMA-15 and maximum stress. However, it was found that the maximum stress of GelMA-15B1 was greatly improved, and that of GelMA-15B10 was about twice that of GelMA-15B1 and about 17 times that of GelMA-15. The compression modulus of the composite hydrogel containing BCP-NP and GelMA-15 is shown in Figure 4b. The compressive modulus was determined by the slope of the elastic region of the stress–strain curve. Like the stress–strain curve, as the ratio of BCP-NP increased, the compression ratio (4, 6, 81, and 93 kPa) was shown, and statistically significant differences were shown in all groups (*p* <0.05). In particular, GelMA-15B1 showed a large increase of about 20 times, compared to GelMA-15, and GelMA-15B10 showed a difference of about 23 times. In addition, this result was shown to be similar to the previous stress–strain data, and the data for each group are shown in Supplementary Materials Table S1.

### 3.5. Thermal Properties

**Figure 5:**
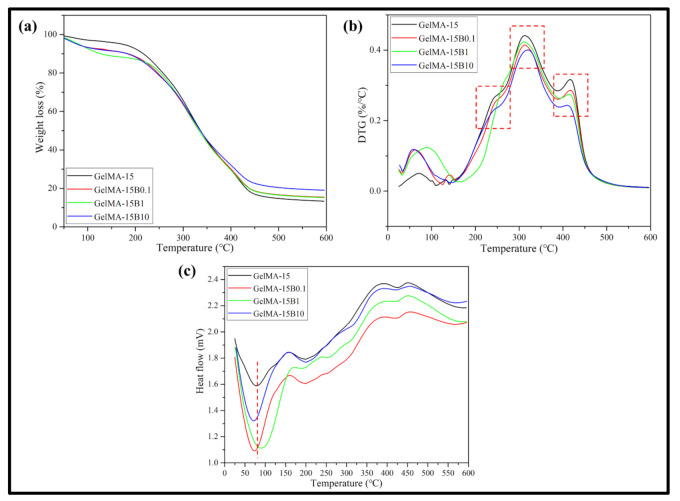
Thermal properties analysis of GelMA-15, GelMA-15B0.1, GelMA-15B1, and GelMA-15B10 hydrogels. (**a**) Thermogravimetric (TG) and (**b**) derived thermogravimetric (DTG) analysis. (**c**) Differential scanning calorimetry (DSC) curve.

The thermal properties of the hydrogel were carried out through a thermal analyzer. The TGA curve shows that the hydrogel began to lose integrity between 50 and 450 and the mass decreased (Figure 5a). In particular, the hydrogel containing BCP-NP showed a reduced mass loss from 350 °C, under the influence of BCP-NP. The final mass loss of all hydrogels was identified as −87% for GelMA-15, −85% for GelMA-15B0.1, −84% for GelMA-15B1, and −80% for GelMA-15B10, respectively. It was confirmed that a large amount of weight loss occurred through the DTG curve (Figure 5b). The first degradation of the hydrogel started between about 50 and 100 °C, and the composite hydrogel containing BCP-NP showed a greater loss than the single hydrogel. However, at 200 to 417 °C, where large weight loss occurs, the composite hydrogel containing BCP-NP showed lower weight loss than GelMA-15. For example, the red square in Figure 5b shows that the composite hydrogel containing BCP-NP has lower mass loss than GelMA-15. The DSC curve showed several endothermic peaks and occurred largely between 50 and 100 °C. (Figure 5c). In particular, the endothermic peak of the composite hydrogel containing BCP-NP moved to a lower temperature than GelMA-15 (90 °C). For example, GelMA-15B10 showed a peak at 75 °C, due to an increase in the BCP-NP content in the hydrogel.

### 3.6. Enzymatic Degradation

**Figure 6:**
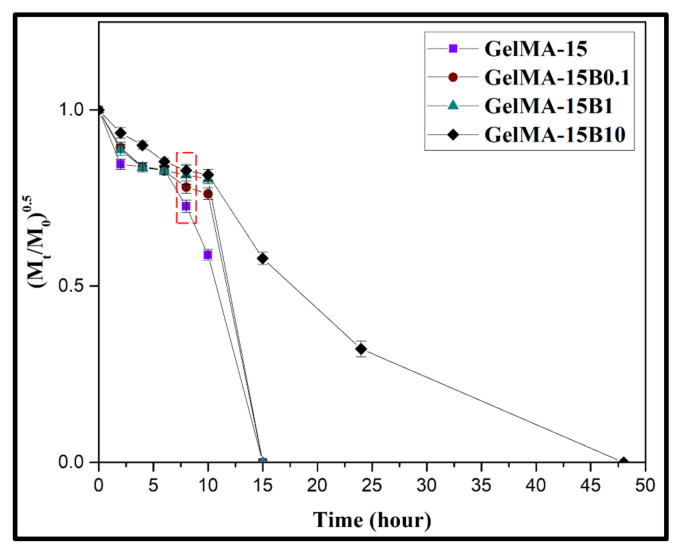
Biodegradation characteristics of GelMA-15, GelMA-15B0.1, GelMA-15B1, and GelMA15-B10 induced by treatment with collagenase type II (25 U/mL^−1^).

Hydrogels using gelatin have been confirmed to be biodegraded by enzymes, because gelatin contains a functional sequence that is stimulated by collagenase [8,25]. Therefore, to investigate the biodegradation effect of the composite hydrogel applied with GelMA and BCP-NP for enzymatic degradation, it was treated with collagenase type Ⅱ. The remaining hydrogel was measured at a predetermined time (2, 4, 6, 8, 10, 15, 24, and 48 h). Figure 6 shows a graph of (M_t_/M_0_)^0.5^ versus t. Moreover, (M_t_/M_0_)^0.5^ represents the amount of degradation for the hydrogel, at the specified time, t, for the hydrogel and composite hydrogel. There was no difference in the degradation rate in all hydrogels, until the initial 2 h. However, from 8 h, GelMA-15 and hydrogel containing BCP-NP showed a large difference in degradation rate. In addition, GelMA-15, GelMA-15B0.1, and GelMA-15B1 groups were all degraded based on 15 h, and only the GelMA-15B10 group showed stability against enzymes for up to 48 h. The breakdown of the hydrogel is affected by the enzymatic cleavage of the GelMA gelatin backbone. However, the application of BCP-NP showed that the binding of the GelMA network and the BCP-NP could delay the enzyme cleavage by linking the chains of GelMA.

### 3.7. Cell Viability against Preosteoblasts

**Figure 7:**
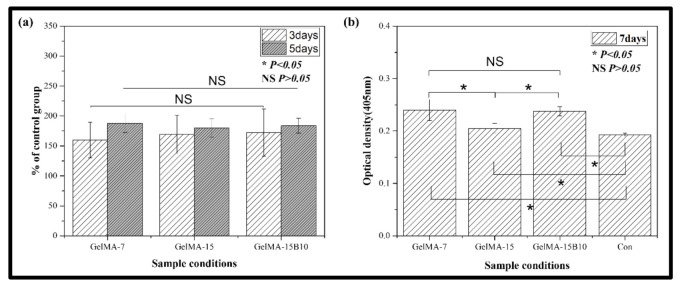
Proliferation (by water-soluble tetrazolium salt (WST)) and differentiation assessment (by alkaline phosphatase (ALP)) of MC3T3-E1 cells on the GelMA-7, GelMA-15, GelMA-15B10, and Control(con) groups after (**a**) three and five days of culture for WST assay and (**b**) seven days of culture for ALP assay.

Various concentrations of GelMA (7% and 15%) and GelMA-15B10 were selected for cell activity evaluation, and a control group in which only cells were dispensed was added for comparison. The results of cell proliferation were expressed in %, based on the control group (Figure 7a). As a result, during the initial three and five days, all groups improved more than 150%, as compared to the control group. However, there was no significant difference in GelMA-7, GelMA-15, and GelMA-15B10 (*p* >0.05). Cellular differentiation was confirmed through the expression of ALP activity (Figure 7b). Similar to the previous cell proliferation results, after seven days of culture, all hydrogel groups showed higher ALP activity than the control group, and showed a statistically significant difference (*p* <0.05). When comparing the hydrogel groups, GelMA-7 and GelMA-15B10 showed significantly increased ALP activity, as compared to GelMA-15 (*p* <0.05). However, there was no significant difference between GelMA-7 and GelMA-15B10 (*p* >0.05).

## 4. Discussion

Synthesis of the substituted GelMA monomer by combining gelatin with MA confirmed the presence of methacrylate bound to the gelatin skeleton through 1H-NMR analysis. In Figure S1, no acrylic protons (6.1 ppm) were identified, and the discovery of GelMA’s typical methacrylate proton C (5.4 ppm) and proton B (5.7 ppm) shows that MA was successfully grafted onto the gelatin skeleton. In addition, it was confirmed that the increase of 1.8 ppm and the decrease of 2.9 ppm, which are reactions of lysine protons in GelMA, were produced when COOH^−^ of MA was combined with NH^−^ of gelatin [26,27].

The photo-crosslinkable hydrogel to which BCP-NP is physically bound was prepared, using a photo-initiator (TEA/VC/Eosin Y) mediating visible light. When exposed to UV rays, cells themselves and their functions are impaired, which may lead to tumors or cancers, so we tried to exclude them from the study [12,28]. Irgacure-2959 could not be activated with visible light because of its low water solubility and limited molar absorption. Although LAP has high water solubility and cellular compatibility, it has low activity in the visible range (405 nm) and high activity in the ultraviolet range (365–385 nm), making it unsuitable for use as an activation initiator in the visible range [29]. In particular, considering the effective wavelength (420–480 nm) of a dental-curing device that can be used clinically in tissue engineering, the possibility of using a cutting type photo-initiator is limited. To solve this problem, Eosin Y, known as an uncut photo-initiator, was used. Eosin Y not only minimizes the safety issues related to UV light, but can also be quickly activated with the effective wavelength used in dental-treatment systems. In addition, TEA and VC were used as co-initiators and monomers, respectively, to aid in free radical photoinitiation [30].

FTIR is known to be the most practical method for comparing organic compounds. The produced hydrogel and nanoparticles were further confirmed through FTIR and XRD analysis. The composite hydrogel was compared with GelMA-15 by selecting GelMA-15B10. The comparison of GelMA-15 and GelMA-15B10 in FTIR was confirmed through the fingerprint area. The fingerprint area is allocated in the area of 500~1500 cm^−1^ and is useful for comparison because it shows a unique pattern for organic compounds [31]. The binding of BCP-NP could be confirmed through the newly created PO stretching band (890 cm^−1^) and phosphate band (500–600 cm^−1^) (Figure 2a). Moreover, to confirm the generation of BCP-NP, comparison with HAP and TCP was conducted. BCP-NP and other nanoparticles also showed different properties (Figure 2b). HAP and TCP showed similar bands, but it was confirmed that only BCP-NP exhibited characteristic bands related to PO and POH [32], which confirmed that the production of BCP-NP was successful. GelMA-15B0.1 showed a wrinkled shape, instead of a hole shape, by mixing a little BCP-NP. However, as a result of observing the surface by using FE-SEM, the presence of BCP-NP could not be confirmed (Figure 3c,d).

The ability to maintain shape and mechanical properties during the material replacement and bone regeneration processes is the most ideal feature of guided bone generation and bone-graft materials. Hence, it is essential to maintain a balance between the degree of material degradation and bone formation. Achievement of improper mechanical strength results in the inability of the material to maintain its shape. Mimicking bone-forming tissues is an important strategy used to prepare biomimetic hydrogels for bone-tissue engineering. Approximately 35% of natural bone is made of organic substances (mainly type 1 collagen), while approximately 65% consists of inorganic substances (nanocrystalline calcium phosphate, HAP, and TCP) [33]. The composite hydrogel group containing BCP-NP showed higher stress, compared to the single hydrogel (Figure 4a). This seems to be because BCP-NP is bound to the hydrogel and acts as a crosslink to increase the strength of the material. The compressive elastic modulus also showed a tendency to increase as the ratio of BCP-NP increased. These results showed a tendency to agree with the previous stress–strain curve. In addition, the increase in the elasticity of the hydrogel can be an index, showing that it can resist external pressure when implanted at a defect site.

The thermal behavior of hydrogel samples for various thermal applications was characterized based on TGA (Figure 5). From the results of TGA and DTG, all hydrogels showed some mass loss up to 100 °C, which corresponds to dehydration, the thermal behavior of GelMA-based hydrogels. It is known that the mass loss of the hydrogel starting at 200 °C (Figure 5a) is mainly due to hydrogen bonds between molecules and the breakdown of molecular side chains of the gel component [34]. The observed thermal behavior of the hydrogel is due to previously published performance, and the addition of BCP nanoparticles to the polymer matrix did not significantly alter the polymer’s thermal properties. This indicates a large weight loss of the entire sample because the decomposition process of GelMA-15 has already ended at about 375 °C, and in the case of the composite hydrogel containing BCP-NP, the degradation process ended at the same temperature as GelMA-15. However, the BCP-NP integration was confirmed due to the result of the remaining mass loss of the sample. Bone-regeneration conditions are determined by the interaction between the cells at the site of the bone defect and the surface of the implant material. When the composite hydrogel is implanted, the immune system will recognize the antigenic determinants provided to the hydrogel, and the immune system will probably reject the transplant [35]. Thus, one of the success factors for hydrogel application is to reduce immune rejection and increase the biocompatibility of biomaterials. The cell compatibility of the hydrogel was confirmed by using mouse-derived preosteoblasts (MC3T3-E1 cells) and cell proliferation and differentiation tests (Figure 7a,b). The higher the concentration of GelMA, the stronger the mechanical properties, but the lower the cell activity. In the previous literature, it was confirmed that a hydrogel with a GelMA concentration of 7–8% exhibits high cellular activity [36]. In this study, a hydrogel with a GelMA concentration of 15% was prepared, and a GelMA concentration of 7% hydrogel was additionally prepared to compare cell activity. Cell proliferation was not significantly different in all hydrogel groups but increased significantly compared to the control group. However, the differentiation of cells by ALP activity showed different results. As reported in the previous literature, GelMA-7 showed better cell differentiation than GelMA-15, and a statistically significant difference was also confirmed. However, GelMA-15B10 showed better cell differentiation than GelMA-15, and there was no significant difference from GelMA-7. It can be seen that BCP-NP contributed to increased cell differentiation. Bone tissue has a hierarchical structure ranging from macroscopic to nanoscale, and many studies have attempted to mimic it. It is also known that hydrogels provide a dynamic environment similar to that of the extracellular matrix [37,38]. In this study, nanosized BCP-NP was contained in a composite hydrogel, and a nano-shaped surface was provided for cell adhesion. BCP-NP is known to stimulate cellular activity by stimulating the proliferation of hMSCs [39] but is expected to sufficiently affect preosteoblasts (MC3T3-E1) and promote cellular activity.

## 5. Conclusions

In this study, a hydrogel applicable to bone-tissue engineering was fabricated, using gelatin methacrylate with excellent biocompatibility, and characteristics were evaluated by applying BCP-NP. According to the analysis, the nano BCP-NP is a mixed form of HA and b-TCP, and characteristic spectra and peaks of BCP-NP were confirmed through XRD and FTIR. The composite hydrogel (GelMA-15B10) improved mechanical properties and increased up to 23 times more than GelMA-15. In addition, through enzymatic degradable analysis, it was confirmed that the composite hydrogel has better durability than the single hydrogel. The visible light initiator and BCP-NP used in the hydrogel production showed good results for preosteoblast cells (MC3T3-E1), and, in particular, BCP-NP improved cell differentiation in the hydrogel. Based on these results, the composite hydrogel to which BCP-NP is applied not only has stability against biodegradation but is expected to be used as a material applicable to the field of bone-tissue engineering in the future.

## Data Availability

The data presented in this study are available in Supplementary Material file.

## Supplementary Materials

The following are available online at https://www.mdpi.com/2079-4991/11/3/617/s1, Figure S1. 1H-NMR spectra of gelatin and gelatin methacryloyl (GelMA) monomer with degree of substitution; Figure S2. The energy-dispersive X-ray (EDX) profile of (a) GelMA-15, (b) GelMA-15B0.1, (c) GelMA-15B1, (d) GelMA-15B10.; Table S1. Maximum stress and Compressive Modulus for each hydrogel groups.

## References

[1] ScSculean A., Nikolidakis D., Nikou G., Ivanovic A., Chapple I.L., Stavropoulos A. (2015). Biomaterials for promoting periodontal regeneration in human intrabony defects: A systematic review. Periodontology 2000.

[2] Kao R.T., Nares S., Reynolds M.A. (2015). Periodontal regeneration–Intrabony defects: A systematic review from the AAP regeneration workshop. J. Periodontol..

[3] Athirasala A., Tahayeri A., Thrivikraman G., França C.M., Monteiro N., Tran V., Ferracane J., Bertassoni L.E. (2018). A dentin-derived hydrogel bioink for 3D bioprinting of cell laden scaffolds for regenerative dentistry. Biofabrication.

[4] Monteiro N., Thrivikraman G., Athirasala A., Tahayeri A., França C.M., Ferracane J.L., Bertassoni L.E. (2018). Photopolymerization of cell-laden gelatin methacryloyl hydrogels using a dental curing light for regenerative dentistry. Dent. Mater..

[5] Nichol J.W., Koshy S.T., Bae H., Hwang C.M., Yamanlar S., Khademhosseini A. (2010). Cell-laden microengineered gelatin methacrylate hydrogels. Biomaterials.

[6] Lin R.-Z., Chen Y.-C., Moreno-Luna R., Khademhosseini A., Melero-Martin J.M. (2013). Transdermal regulation of vascular network bioengineering using a photopolymerizable methacrylated gelatin hydrogel. Biomaterials.

[7] Fang X., Xie J., Zhong L., Li J., Rong D., Li X., Ouyang J. (2016). Biomimetic gelatin methacrylamide hydrogel scaffolds for bone tissue engineering. J. Mater. Chem. B.

[8] Xiao S., Zhao T., Wang J., Wang C., Du J., Ying L., Lin J., Zhang C., Hu W., Wang L. (2019). Gelatin methacrylate (GelMA)-based hydrogels for cell transplantation: An effective strategy for tissue engineering. Stem Cell Rev. Rep..

[9] Luo Z., Sun W., Fang J., Lee K., Li S., Gu Z., Dokmeci M.R., Khademhosseini A. (2019). Biodegradable gelatin methacryloyl microneedles for transdermal drug delivery. Adv. Healthc. Mater..

[10] Eslami M., Vrana N.E., Zorlutuna P., Sant S., Jung S., Masoumi N., Khavari-Nejad R.A., Javadi G., Khademhosseini A. (2014). Fiber-reinforced hydrogel scaffolds for heart valve tissue engineering. J. Biomater. Appl..

[11] Cha C., Shin S.R., Gao X., Annabi N., Dokmeci M.R., Tang X., Khademhosseini A. (2014). Controlling mechanical properties of cell-laden hydrogels by covalent incorporation of graphene oxide. Small.

[12] Wang Z., Abdulla R., Parker B., Samanipour R., Ghosh S., Kim K. (2015). A simple and high-resolution stereolithography-based 3D bioprinting system using visible light crosslinkable bioinks. Biofabrication.

[13] Cidonio G., Alcala-Orozco C.R., Lim K.S., Glinka M., Mutreja I., Kim Y.-H., Dawson J.I., Woodfield T.B., Oreffo R.O. (2019). Osteogenic and angiogenic tissue formation in high fidelity nanocomposite Laponite-gelatin bioinks. Biofabrication.

[14] Thakur T., Xavier J.R., Cross L., Jaiswal M.K., Mondragon E., Kaunas R., Gaharwar A.K. (2016). Photocrosslinkable and elastomeric hydrogels for bone regeneration. J. Biomed. Mater. Res. Part A.

[15] Paknejad M., Emtiaz S., Rokn A., Islamy B., Safiri A. (2008). Histologic and histomorphometric evaluation of two bone substitute materials for bone regeneration: An experimental study in sheep. Implant Dent..

[16] García-Gareta E., Coathup M.J., Blunn G.W. (2015). Osteoinduction of bone grafting materials for bone repair and regeneration. Bone.

[17] Giannoudis P.V., Dinopoulos H., Tsiridis E. (2005). Bone substitutes: An update. Injury.

[18] Lan W., Zhang X., Xu M., Zhao L., Huang D., Wei X., Chen W. (2019). Carbon nanotube reinforced polyvinyl alcohol/biphasic calcium phosphate scaffold for bone tissue engineering. RSC Adv..

[19] Nie L., Wu Q., Long H., Hu K., Li P., Wang C., Sun M., Dong J., Wei X., Suo J. (2019). Development of chitosan/gelatin hydrogels incorporation of biphasic calcium phosphate nanoparticles for bone tissue engineering. J. Biomater. Sci. Polym. Ed..

[20] Faruq O., Kim B., Padalhin A.R., Lee G.H., Lee B.-T. (2017). A hybrid composite system of biphasic calcium phosphate granules loaded with hyaluronic acid–Gelatin hydrogel for bone regeneration. J. Biomater. Appl..

[21] Zhao X., Lang Q., Yildirimer L., Lin Z.Y., Cui W., Annabi N., Ng K.W., Dokmeci M.R., Ghaemmaghami A.M., Khademhosseini A. (2016). Photocrosslinkable gelatin hydrogel for epidermal tissue engineering. Adv. Healthc. Mater..

[22] Chen Y., Li J., Kawazoe N., Chen G. (2017). Preparation of dexamethasone-loaded calcium phosphate nanoparticles for the osteogenic differentiation of human mesenchymal stem cells. J. Mater. Chem. B.

[23] Li X., Chen S., Li J., Wang X., Zhang J., Kawazoe N., Chen G. (2016). 3D culture of chondrocytes in gelatin hydrogels with different stiffness. Polymers.

[24] Chen Y., Kawazoe N., Chen G. (2018). Preparation of dexamethasone-loaded biphasic calcium phosphate nanoparticles/collagen porous composite scaffolds for bone tissue engineering. Acta Biomater..

[25] van den Steen P.E., Dubois B., Nelissen I., Rudd P.M., Dwek R.A., Opdenakker G. (2002). Biochemistry and molecular biology of gelatinase B or matrix metalloproteinase-9 (MMP-9). Crit. Rev. Biochem. Mol. Biol..

[26] Hu X., Ma L., Wang C., Gao C. (2009). Gelatin hydrogel prepared by photo-initiated polymerization and loaded with TGF-β1 for cartilage tissue engineering. Macromol. Biosci..

[27] Claaßen C., Claaßen M.H., Truffault V., Sewald L., Tovar G.n.E., Borchers K., Southan A. (2018). Quantification of substitution of gelatin methacryloyl: Best practice and current pitfalls. Biomacromolecules.

[28] Sinha R.P., Häder D.-P. (2002). UV-induced DNA damage and repair: A review. Photochem. Photobiol. Sci..

[29] Shih H., Lin C.C. (2013). Visible-light-mediated thiol-Ene hydrogelation using eosin-Y as the only photoinitiator. Macromol. Rapid Commun..

[30] Noshadi I., Hong S., Sullivan K.E., Sani E.S., Portillo-Lara R., Tamayol A., Shin S.R., Gao A.E., Stoppel W.L., Black L.D. (2017). In vitro and in vivo analysis of visible light crosslinkable gelatin methacryloyl (GelMA) hydrogels. Biomater. Sci..

[31] Choi J.B., Jang Y.S., Byeon S.M., Jang J.H., Kim Y.K., Bae T.S., Lee M.H. (2019). Effect of composite coating with poly-dopamine/PCL on the corrosion resistance of magnesium. Int. J. Polym. Mater. Polym. Biomater..

[32] Touny A., Saleh M. (2018). Fabrication of biphasic calcium phosphates nanowhiskers by reflux approach. Ceram. Int..

[33] Pina S., Oliveira J.M., Reis R.L. (2015). Natural-based nanocomposites for bone tissue engineering and regenerative medicine: A review. Adv. Mater..

[34] Liu X., Song T., Chang M., Meng L., Wang X., Sun R., Ren J. (2018). Carbon nanotubes reinforced maleic anhydride-modified xylan-g-poly (N-isopropylacrylamide) hydrogel with multifunctional properties. Materials.

[35] Ramay H.R., Zhang M. (2004). Biphasic calcium phosphate nanocomposite porous scaffolds for load-bearing bone tissue engineering. Biomaterials.

[36] Lee D., Choi E.J., Lee S.E., Kang K.L., Moon H.-J., Kim H.J., Youn Y.H., Heo D.N., Lee S.J., Nah H. (2019). Injectable biodegradable gelatin-methacrylate/β-tricalcium phosphate composite for the repair of bone defects. Chem. Eng. J..

[37] Luo Y., Lode A., Wu C., Chang J., Gelinsky M. (2015). Alginate/nanohydroxyapatite scaffolds with designed core/shell structures fabricated by 3D plotting and in situ mineralization for bone tissue engineering. ACS Appl. Mater. Interfaces.

[38] Zhao C., Xia L., Zhai D., Zhang N., Liu J., Fang B., Chang J., Lin K. (2015). Designing ordered micropatterned hydroxyapatite bioceramics to promote the growth and osteogenic differentiation of bone marrow stromal cells. J. Mater. Chem. B.

[39] Chen Y., Wang J., Zhu X., Tang Z., Yang X., Tan Y., Fan Y., Zhang X. (2015). Enhanced effect of β-tricalcium phosphate phase on neovascularization of porous calcium phosphate ceramics: In vitro and in vivo evidence. Acta Biomater..

