# Supplementary material for: Fabrication and Characterization of Biodegradable Gelatin Methacrylate/Biphasic Calcium Phosphate Composite Hydrogel for Bone Tissue Engineering
Source: Nanomaterials (Basel). 2021 Mar 2;11(3):617. doi: 10.3390/nano11030617 (PMC7999599; doi:10.3390/nano11030617)
Supplement: Supplementary file 1 [file nanomaterials-11-00617-s001.pdf]

Supplementary Material

Fabrication and Characterization of Biodegradable Gelatin Methacrylate/Biphasic Calcium Phosphate Composite Hydrogel for Bone Tissue Engineering

Ji-Bong Choi [1,†], Yu-Kyoung Kim[1,†], Seon-Mi Byeon [2], Jung-Eun Park [1], Tae-Sung Bae [1], Yong-Seok Jang [1,*] and Min-Ho Lee [1,*]

[1] Department of Dental Biomaterials, Institute of Biodegradable Materials, School of Dentistry, Jeonbuk National University, Jeonju-si, jeollabuk-do, Korea, 54896; submissi@naver.com (J.-B.C.); yk0830@naver.com (Y.-K.K.); pje312@naver.com (J.-E.P.); bts@jbnu.ac.kr (T.-S.B.)

[2] Dental Clinic of Ebarun, Suncheon-si, Jeollanam-do, Korea, 57999; sumse1205@naver.com

* Correspondence: Yjang@jbnu.ac.kr (Y.-S.J.); mh@jbnu.ac.kr (M.-H.L.)

† These authors contributed equally to the paper.

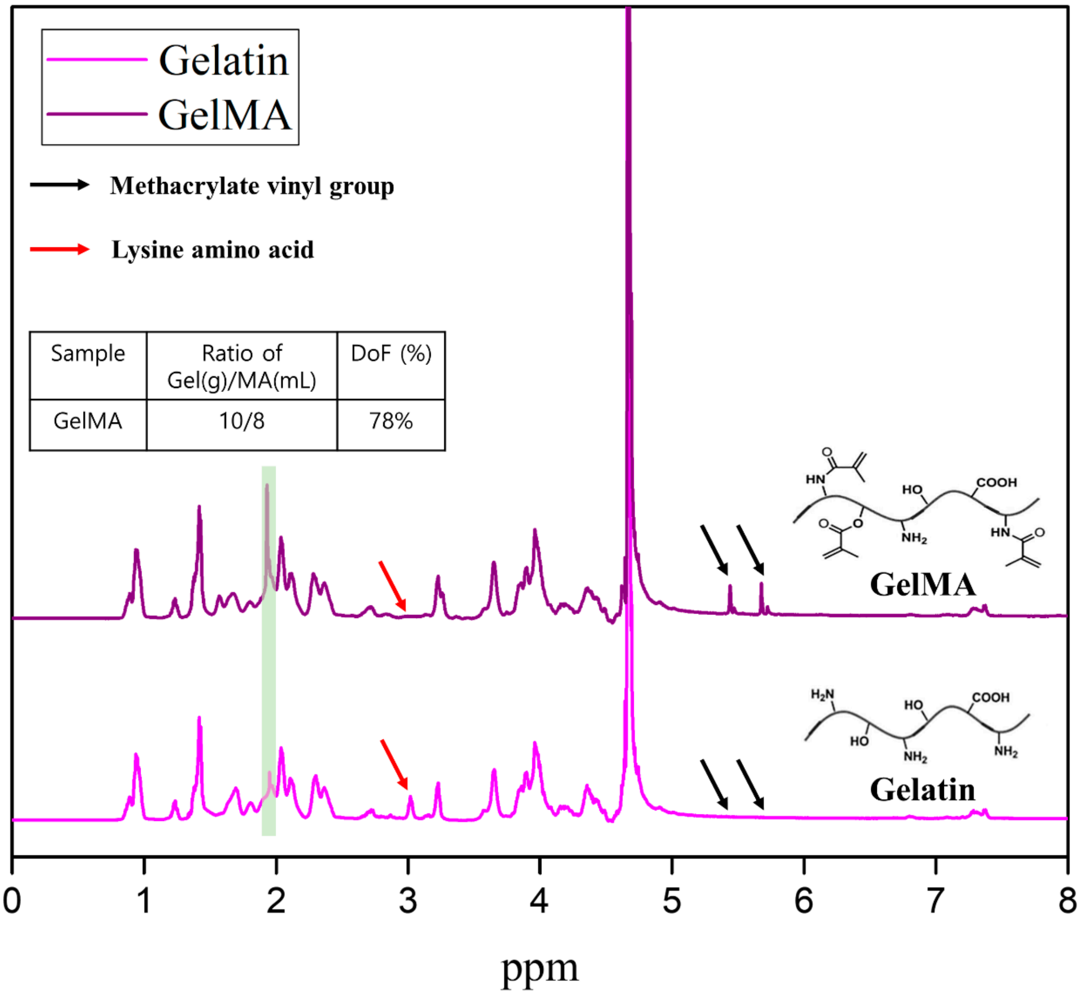

Figure S1. 1H-NMR spectra of gelatin and gelatin methacryloyl (GelMA) monomer with degree of substitution.

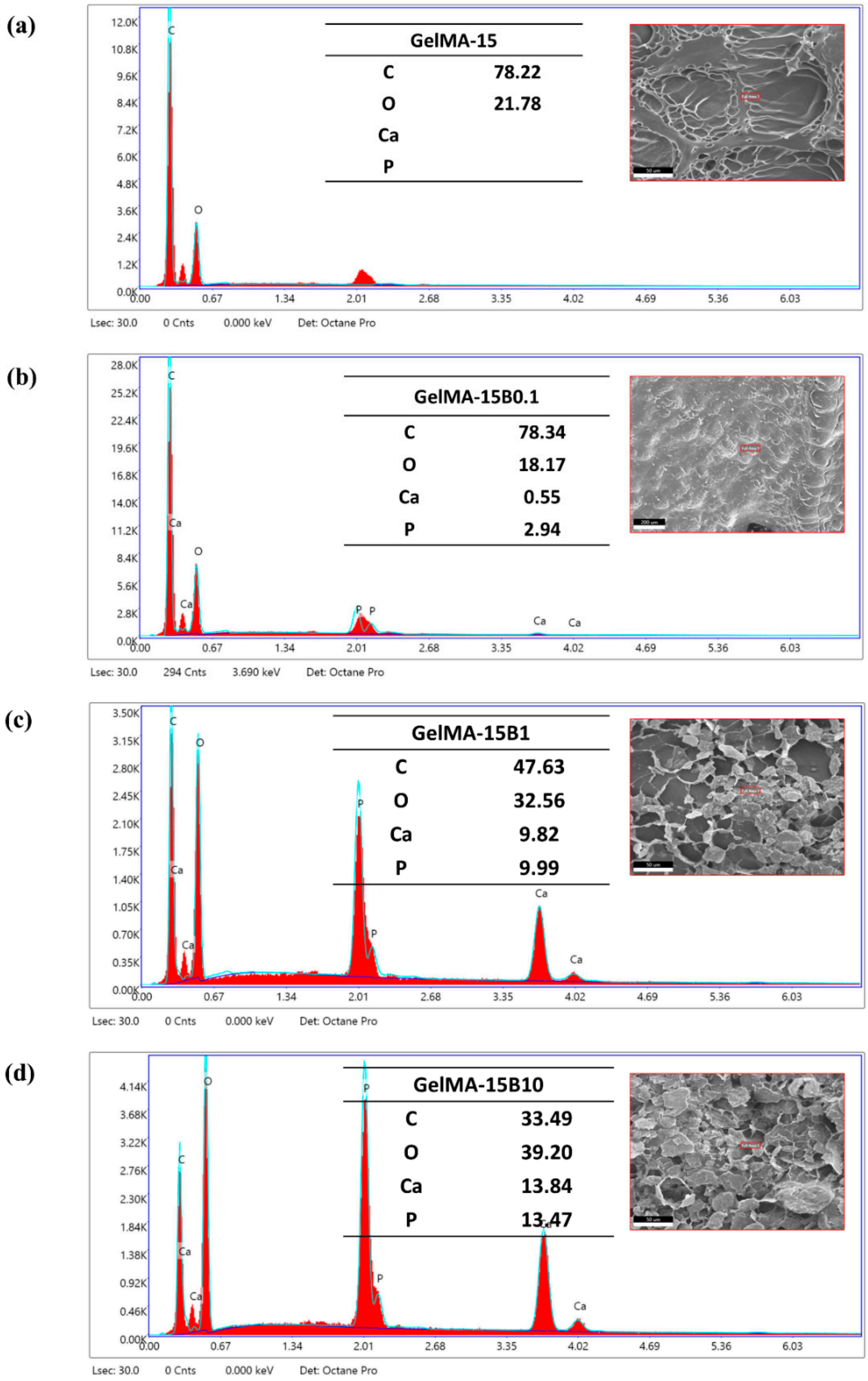

Figure S2. The energy-dispersive X-ray (EDX) profile of (**a**) GelMA-15, (**b**) GelMA-15B0.1, (**c**) GelMA-15B1, (**d**) GelMA-15B10

Table S1. Maximum stress and Compressive Modulus for each hydrogel groups.

**Groups**	Maximum stress(kPa)	Compressive Modulus(kPa)
GelMA-15	25.38 ± 0.30	4.13 ± 0.07
GelMA-15B0.1	55.78 ± 0.61	6.69 ± 0.02
GelMA-15B1	222.79 ± 0.59	81.69 ± 0.30
GelMA-15B10	442.26 ± 1.29	93.66 ± 0.44
